# Sexually Dimorphic Body Color Is Regulated by Sex-Specific Expression of *Yellow* Gene in Ponerine Ant, *Diacamma* Sp

**DOI:** 10.1371/journal.pone.0092875

**Published:** 2014-03-25

**Authors:** Satoshi Miyazaki, Yasukazu Okada, Hitoshi Miyakawa, Gaku Tokuda, Richard Cornette, Shigeyuki Koshikawa, Kiyoto Maekawa, Toru Miura

**Affiliations:** 1 Laboratory of Ecological Genetics, Graduate School of Environmental Science, Hokkaido University, Sapporo, Japan; 2 Graduate School of Science and Engineering, University of Toyama, Toyama, Japan; 3 Graduate School of Arts and Sciences, University of Tokyo, Meguro, Tokyo, Japan; 4 Okazaki Institute for Integrative Bioscience, National Institute of Basic Biology, Okazaki, Japan; 5 Center of Molecular Biosciences, University of the Ryukyu, Okinawa, Japan; 6 Insect Mimetics Research Unit, National Institute of Agrobiological Sciences, 1-2 Owashi, Tsukuba, Ibaraki, Japan; 7 Laboratory of Molecular Biology, University of Wisconsin-Madison, Madison, Wisconsin, United States of America; U. Kentucky United States of America

## Abstract

Most hymenopteran species exhibit conspicuous sexual dimorphism due to ecological differences between the sexes. As hymenopteran genomes, under the haplodiploid genetic system, exhibit quantitative differences between sexes while remaining qualitatively identical, sexual phenotypes are assumed to be expressed through sex-specific gene usage. In the present study, the molecular basis for expression of sexual dimorphism in a queenless ant, *Diacamma* sp., which exhibits a distinct color dimorphism, was examined. Worker females of the species appear bluish-black, while winged males exhibit a yellowish-brown body color. Initially, observations of the pigmentation processes during pupal development revealed that black pigmentation was present in female pupae but not in males, suggesting that sex-specific melanin synthesis was responsible for the observed color dimorphism. Therefore, five orthologs of the genes involved in the insect melanin synthesis (*yellow*, *ebony*, *tan, pale* and *dopa decarboxylase*) were subcloned and their spatiotemporal expression patterns were examined using real-time quantitative RT-PCR. Of the genes examined, *yellow*, which plays a role in black melanin synthesis in insects, was expressed at higher levels in females than in males throughout the entire body during the pupal stage. RNA interference of *yellow* was then carried out in order to determine the gene function, and produced females with a more yellowish, brighter body color similar to that of males. It was concluded that transcriptional regulation of *yellow* was responsible for the sexual color dimorphism observed in this species.

## Introduction

In many animal species, sexual characteristics are often distinctive between males and females, directly or indirectly related to reproductive activities. These sex differences, generally known as sexual dimorphism, are suggested to have evolved through either sexual selection or intraspecific niche divergence [1]. Social hymenopterans, groups in which females engage in social behavior but males do not [2], [3], show a conspicuous sexual dimorphism related to the ecological differences between sexes [4]. In these groups, sex is determined by haplodiploidy; fertilized eggs develop into females, while unfertilized eggs develop into males [5]. Since this system does not require sex chromosomes, no sex-specific component should exist within hymenopteran genomes. Therefore, the sexual dimorphism in social hymenopterans must derive from qualitatively identical genomes; however, the proximate mechanism for this sexual dimorphism has not yet been determined [4].

Sex determination in insects is generally controlled by a cascade of splicing factors, which are alternatively spliced in a sex-specific manner. Although upstream genes in this cascade differ among species, the final step is shared across a diverse array of insect taxa; the cascade eventually affects the sex-specific splicing of the most downstream gene, *doublesex* (*dsx*) [6]. The resulting sex-specific Dsx protein acts as a switch for sexual differentiation by regulating downstream gene expression [7]. In Hymenoptera, *dsx* also acts as a sexual switch [8], [9]. Therefore, some sets of genes shared by both sexes must be expressed in a sex-specific manner through the action of *dsx*, resulting in conspicuous sexual differences. In the present study, the downstream genes of the cascade, which are responsible for conspicuous sexually dimorphic characters, were examined in a queenless ponerine ant, *Diacamma* sp. (Formicidae; Ponerinae).

**Figure 1:**
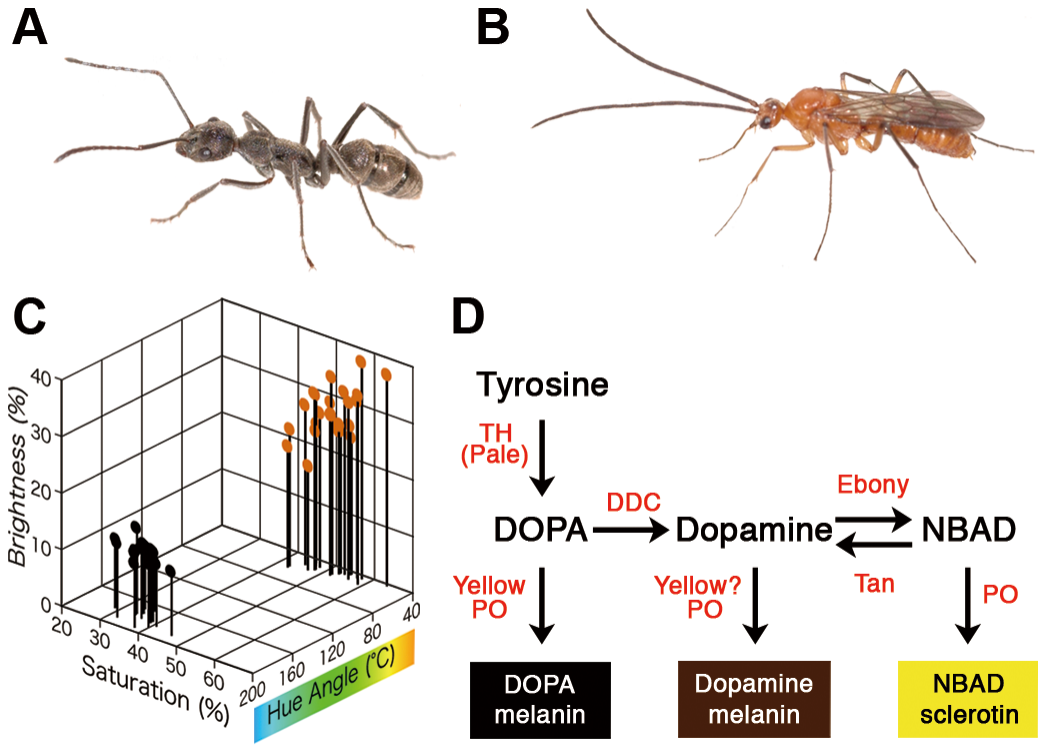
Sexually dimorphic pigmentation in *Diacamma* sp. Females exhibit a bluish-black color (A), while males appear yellowish-brown (B). Adult body color was evaluated in females (black dots) and males (orange dots) using a HSB (hue, saturation, and brightness) color model (C). Hypothetical pathway for synthesizing melanins (D). Melanin related pigments are represented by boxes, whose colors correspond to the hypothesized colors of each respective pigment. Pigment precursors and enzymes are represented in black and red lettering, respectively. (TH; Tyrosine Hydroxylase, DDC; Dopa Decarboxylase, PO; Phenoloxidase, DOPA; Dihydroxypheylalanine, NBAD; N-β-alanyldopamine)

*Diacamma* sp. possesses various sex-specific traits (Fig. 1A and B) that exhibit extreme differences compared to those observed in other hymenopteran species [10]. In this species, body color is one of the most distinctive sexual traits; females exhibit a bluish-black color, whereas males possess a yellowish-brown body (Fig. 1A-C). This color difference is shared by all species in the genus with only a few exceptions [11]. In general, insect body color largely depends on the composition of pigments (i.e., melanin, pterin and ommochromes) within the cuticle and/or epidermis [12]. Melanin pigments appear black, gray, or brown [13] and they are among the most common pigments in a wide range of animal taxa [14], [15]. The developmental, genetic and biochemical bases of melanin synthesis have been extensively studied in Dipteran, Lepidopteran and Coleopteran species [13], [16], [17]. In melanin synthesis (Fig. 1D), pigment precursors (tyrosine, DOPA, dopamine and NBAD) are incorporated from the hemolymph into epidermal cells, and there, the relative amounts of pigment precursors are determined by the action of the enzymes Tyrosine hydroxylase (TH), DOPA decarboxylase (DDC), Ebony, and Tan. Subsequently, Yellow and some phenoloxidases (PO) catalyze the conversion of precursors into three types of pigments (DOPA melanin, dopamine melanin and NBAD sclerotin).

In the present study, in order to understand the developmental and molecular bases responsible for the conspicuous sexual dimorphism in Hymenoptera, it was determined that sex-specific usage of the melanin synthesis pathway contributed to sexually dimorphic coloration in *Diacamma* sp. Initially, pigmentation processes in both sexes were externally and histologically observed during pupal development. Then, expression profiles of pigmentation genes belonging to the melanin synthesis pathway were elucidated using real-time quantitative RT-PCR. qPCR determined that *yellow* was expressed in a female-specific manner during pupal development. Finally, functional analysis using *yellow* RNAi confirmed that *yellow* expression in females was responsible for the observed sexually dimorphic coloration.

## Results

### Pigmentation processes during the pupal stage

**Figure 2:**
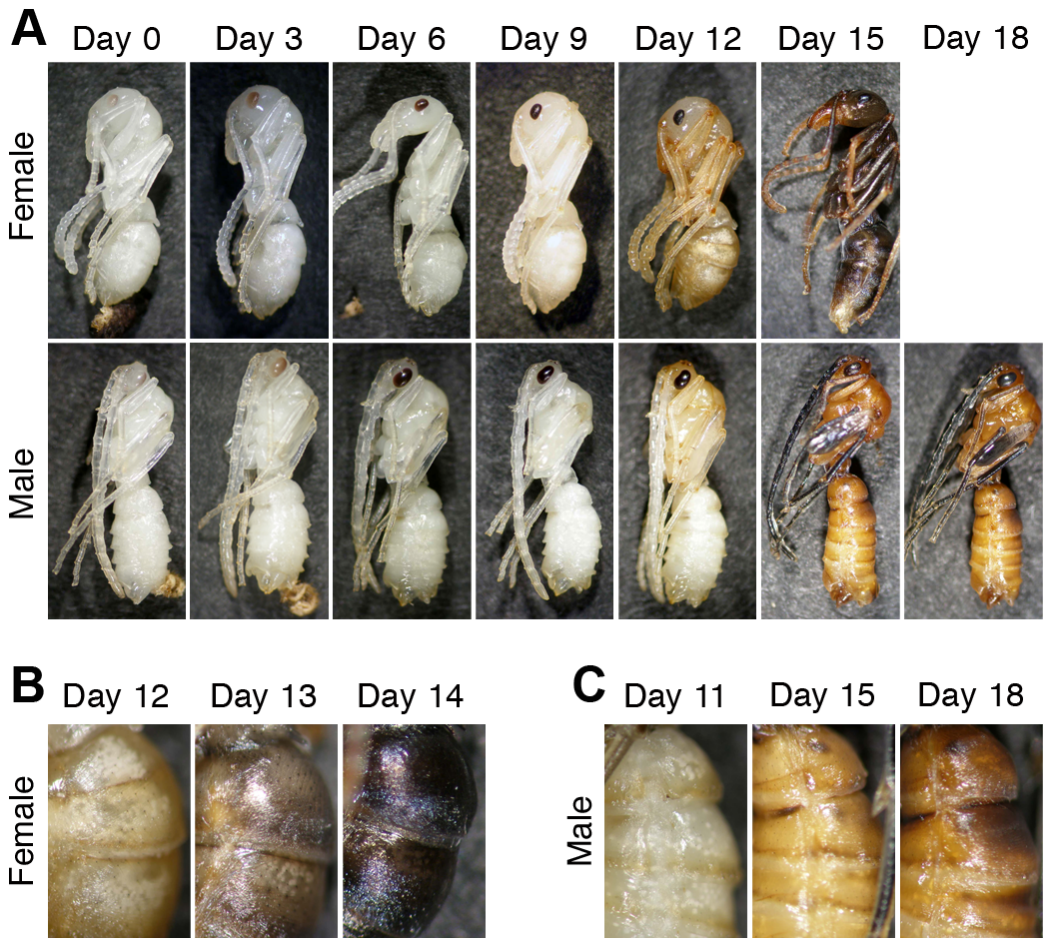
Pigmentation process during pupal development in females and males. Cuticular pigmentation progressed in a sex-specific manner (A). In female pupae, body color gradually turned from white to light-brown, to gray-brown, and finally to black (B). In male pupae, body color turned yellowish-brown, via light-brown phase (C).

The pupal period differed between sexes, lasting 14.6±1.1 days in females (mean ± SD, n = 46) and 17.5±1.8 days in males (n = 26). Immediately after pupation, cuticles contained no pigment, with pigmentation being observed first in the compound eye (Fig. 2A). Sex differences were not observed in the eye pigmentation process. Although cuticular pigmentation of the remainder of the body (hereafter referred to as “pigmentation”) began at the posterior margins of trunk segments and appendages after day 9 in both sexes, subsequent pigmentation processes exhibited sexual differences (Fig. 2A). Pigmentation occurred in the adult cuticle, which was formed under the pupal cuticle (Fig. S1, Text S1). In females, overall color was pale brown by day 11–12, light gray-brown by day 12–13, and brownish-black by day 14 (Fig. 2B). At this stage, pigmentation in females was weaker in the mouthparts, antennal segments, tarsi, and genitalia than in other parts. Pigmentation was complete within a week subsequent to eclosion, at which point female body color was black. In males, the majority of the body was light yellowish-brown by day 13–15, while some parts, e.g., the wing buds, antennae, and tarsus of legs, were darkly pigmented or became black by day 15 (Fig. 2A). At day 16, pigmentation neared completion and male body color became yellowish-brown (Fig. 2A and C).

These observations demonstrated that a large amount of black pigments (probably melanin) were synthesized in female pupae but not in males, and that brown pigments were synthesized in both sexes. In addition, histological observations revealed a sex-specific pattern of pigment accumulation, in which brownish-black pigments and lighter brown pigments accumulated in female and male cuticles, respectively (Fig. S1, Text S1).

### Expression levels of five pigmentation genes during pupal development

**Figure 3:**
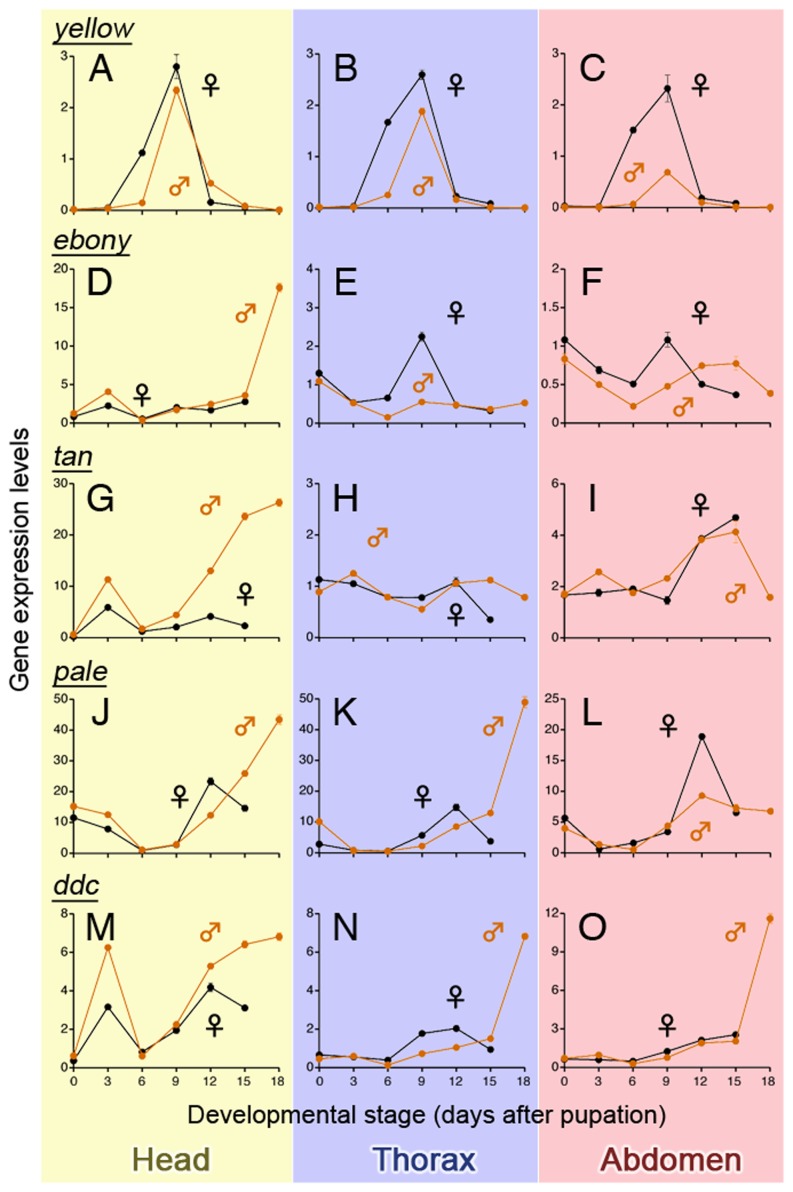
Expression profiles of five pigmentation genes during pupal development of females (black lines) and males (orange lines). Expression levels of *yellow*, *ebony*, *tan*, *dopa decarboxylase* (*ddc*), and *pale* in the head, thorax, and abdomen of each sex were quantified through quantitative RT-PCR. Expression levels were normalized to those of *28S rRNA*. Mean ± SD, n = 3 (technical triplication).

In order to determine the expression patterns of pigmentation genes, fragments of five gene orthologs, i.e. *pale* (encoding TH), *ddc*, *yellow*, *ebony* and *tan*, were subcloned and sequenced (primer designs, lengths of subcloned fragments and accession numbers are shown in Table S1). Two types of *pale* ortholog isoforms with different lengths (532 and 718 bps) were obtained and the present study focused on the long type isoform that was suggested to be involved in insect melanization [18], [19]. Real-time qPCR revealed that *yellow* exhibited a single-peak expression pattern during the pupal period while the other four genes exhibited double- or triple-peak expression patterns (Fig. 3). The expression peak of *yellow* in both sexes occurred at day 9, just prior to the commencement of pigmentation. Expression levels of the *yellow* gene at almost all developmental stages and in each body part were greater in females than in males (Fig. 3A-C). Expression levels of *yellow* in the abdominal cuticle of day 9 pupae were 12.1 and 0.7 times greater than in other abdominal tissues examined in females and males, respectively (Fig. S2A). For *ebony*, *tan*, and *pale*, expression levels in both sexes varied among body parts (Fig. 3D-L). Although *ddc* expression peaks were higher throughout the body of males than in females (Fig. 3M-O), these male-biased expressions were first observed at day 18 when sexually dimorphic pigmentation had almost been established (Fig. 2A). The expression levels of these four genes (i.e. *ebony*, *tan*, *pale*, and *ddc*) in the abdominal cuticle were up to 1.86 times higher and occasionally lower than in other abdominal tissues, regardless of sex (Fig. S2B-E). This result was contrastive to that of *yellow* in female cuticles, in which the expression level was approximately 12 times greater than in the other abdominal tissues (Fig. S2A).

Genes responsible for sexual color dimorphism must be expressed in a sex-specific pattern, and these sex-specific patterns should be similar throughout the body, as the observed color difference between males and females of *Diacamma* sp. was similar throughout the body (Fig. 1A and B). Of the pigmentation genes examined, the expression patterns of *yellow* were most consistent with these observations.

### Functional analysis of *yellow* gene using RNA interference

**Figure 4:**
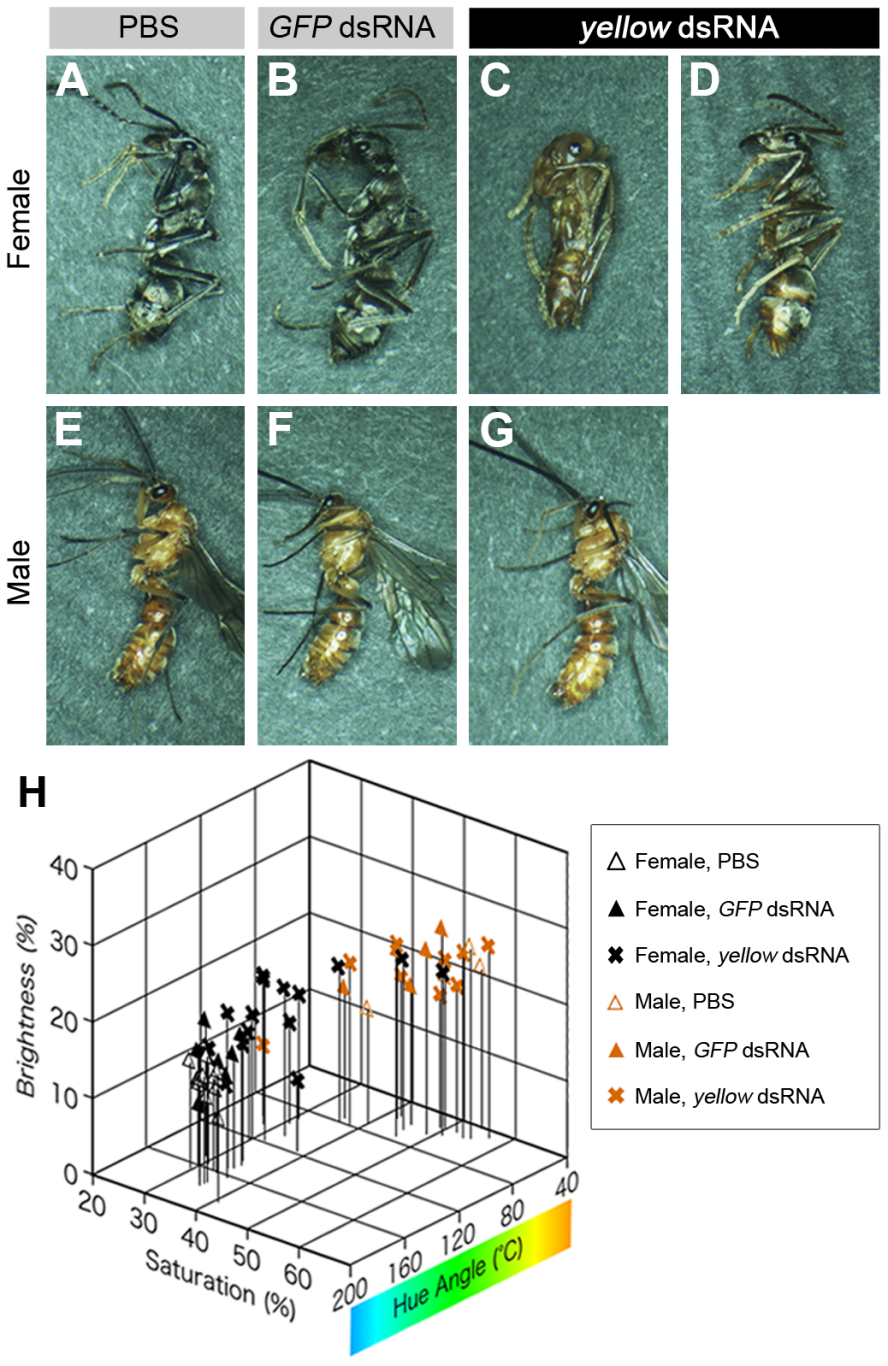
Functional analysis of *yellow* through RNAi. Female and male pupae were injected twice with 1 μl of PBS (A and E) or 10 μg of either *GFP* (B and F) or *yellow* (C, D and G) dsRNA in 1 μl of PBS. Pupae injected with PBS or *GFP* dsRNA exhibited normal body color (A, B, E and F). Injection with *yellow* dsRNA affected pigmentation in females (C and D) but not in males (G). The effect of *yellow* RNAi varied among individual females. One female exhibited strongly impaired black pigmentation throughout the body (C), some exhibited only partially or weakly reduced coloration (D), while others exhibited an almost normal body color. The effect of RNAi on body color was evaluated using a HSB color model (H). The hue angle of *yellow*-RNAi females was significantly decreased and their brightness was significantly increased (see Fig. S3).

In order to elucidate the function of *yellow* in sexually dimorphic pigmentation, pupal RNAi was performed to knockdown gene function. In pupal RNAi experiments, synthesized double-stranded RNA (dsRNA) of *yellow* was injected into pupae at day 0 and 6 (see Materials and Methods), while injection buffer (PBS) or *GFP* (*green fluorescent protein*)-dsRNA was injected in control experiments. All females injected with PBS, *GFP*-, or *yellow*-dsRNA were alive and the majority of them eclosed normally (Table 1). PBS-injected and *GFP*-dsRNA-injected females (n = 17 and 14, respectively) exhibited black body color (Fig. 4A and B) similar to that of untreated females (Fig. 1A). Of the 16 females injected with *yellow* dsRNA, one exhibited a brownish color throughout the body (Fig. 4C), 11 appeared brownish in some body parts, e.g., on the lateral sides of the abdomen, at the distal segments of the appendages, and in the coxae (Fig. 4D), and 4 exhibited normal coloration. Furthermore, the hue angle of body color in *yellow*-RNAi females was significantly lower than in PBS-injected and *GFP*-dsRNA-injected females (*p*<0.01, Tukey’s test), and the brightness of *yellow*-RNAi females was significantly higher than that of both PBS-injected females (*p*<0.01, Tukey’s test) and *GFP* dsRNA-injected females (*p*<0.05, Tukey’s test) (Fig. 4H, Fig. S3). The majority of males used in each treatment were alive, eclosed and exhibited normal body coloration (Table 1, Fig. 4E-G). No color parameter in experimental males significantly differed from that of control males in RNAi experimentation (*p*>0.05, Tukey’s test, Fig. 4H, Fig. S3). In addition, *yellow* RNAi repressed *yellow* expression levels in females, but did not do so in males (Fig. S4, Text S1).

**Table 1:** Effects of RNAi experiments on mortality and eclosion.

Sex	Treatmemt	Eclosed		Failed in eclosion	Dead
female	PBS (n = 17)	17	0		0
	*GFP* dsRNA (n = 16)	14	2		0
	*yellow* dsRNA (n = 16)	14	2		0
male	PBS (n = 11)	8	1		2
	*GFP* dsRNA (n = 10)	7	2		1
	*yellow* dsRNA (n = 20)	12	2		6

## Discussion

### Mechanisms and ecological significance of sexually dimorphic pigmentation in *Diacamma* sp

Observations of the pigmentation processes during pupal development suggested that sex-specific black melanin synthesis in the adult cuticle was responsible for the sexually dimorphic coloration in *Diacamma* sp. Spatiotemporal expression patterns of pigmentation genes indicated that *yellow* was abundant in female cuticles immediately prior to pigmentation. Furthermore, functional analyses by RNAi confirmed that *yellow* played an important role in development of the black pigmentation observed in females. The *yellow* gene is known to contribute to black melanin synthesis [20]; however, it is controversial whether Yellow catalyzes only the conversion of DOPA into DOPA melanin [13], [21]–[23] or also that of dopamine into dopamine melanin [20], [24]. Since the functions of pigmentation genes are thought to be well conserved in insects [13], it was suggested that female-specific *yellow* expression in *Diacamma* sp. regulates black pigmentation in females through the DOPA melanin synthesis, and possibly through the dopamine melanin synthesis as well. In addition, the present study determined that *ddc* was abundant in males during the later pupal stages (Fig. 3M-O). *ddc* is typically expressed during the later stages of insect pigmentation, and is known to catalyze the conversions of DOPA into dopamine [25]–[27]. Dopamine is further converted not only into dopamine melanin, but also into NBAD, which can be a source of NBAD sclerotin that results in yellow or reddish-brown coloration [21], [25]. Therefore, male-specific *ddc* expression could play a role in the yellowish-brown coloration exhibited by males. Unlike *yellow* and *ddc*, no obvious sexual difference existed in the expression patterns of *ebony*, *tan*, and *pale* (Fig. 3D-L); however, these genes are likely to possess well-conserved functions with respect to pigmentation [13]. These results suggested that *ebony*, *tan*, and *pale* were involved in pigmentation itself but did not contribute to the sexual differences observed in *Diacamma* sp. Results of the present study indicated that some genes (e.g., *yellow* and *ddc*) constituting the melanin synthesis pathway were differentially expressed in males and females, which resulted in the sexually dimorphic pigmentation patterns observed in *Diacamma* sp.

Although the adaptive significance of sexually dimorphic pigmentation has not yet been explained, it was believed to reflect their life-history differences between sexes. Female adults work both inside and outside the nest for periods of greater than 200 days [28], while males disperse at 3–4 days after eclosion, inseminate a female from another colony, and then die soon after mating [11], [29]. Therefore, long-lived and active females may require cuticular hardening, which incurs greater energetic costs, more than short-lived males. Since melanins contribute not only to pigmentation, but also to cuticular sclerotization [30], heavily melanized cuticles would be more advantageous in females than in males. Although the benefit to males by possessing the less-melanized cuticles remains unknown, this sex-specific usages of the melanin synthesis pathway are suggested to have been selected for due to these differences in life-history traits.

### Sex-specific gene usage in sexual differentiation

Recent studies using microarrays have provided evidence that gene expression patterns differ between sexes during development in both fire ants [31], [32] and vespine wasps [33]. These studies inferred the importance of sex-specific gene expression for sex differentiation in social hymenopterans, but did not demonstrate how such gene expression contributed to their conspicuous sexual dimorphism. The present study focused on a sexually dimorphic trait and demonstrated that expression of this trait was regulated by the sex-specific expression of a genetic pathway shared by both sexes. Therefore, the present study suggested that sex-specific gene usage served as a major mechanism resulting in the conspicuous sexual dimorphism observed in *Diacamma* sp. and other hymenopterans.

### Regulatory mechanism in the expression of a sexually-dimorphic trait

In the majority of insect species, expression of sexual characters is believed to be controlled by *dsx* [34]–[37], the most downstream factor in the sex-determining gene cascade, through either direct or indirect transcriptional regulation of target genes [38]–[40]. In the case of *Drosophila melanogaster*, a species in which males alone exhibit darkly pigmented posterior abdominal segments, *yellow* expression is regulated by a complex genetic interaction among Bric-à-brac (Bab, a transcription factor), Abdominal-B (Abd-B) Hox protein, and Dsx. In the posterior abdominal segments of females, Abd-B and a female-isoform of Dsx directly activate *bab* expression, and then Bab represses *yellow* expression [39], [41]. In those of males, Abd-B and a male-isoform of Dsx repress *bab* expression, resulting in the male- and segment-specific patterns of *yellow* expression [39], [41]. Suppressive regulations of *bab* with sexually dimorphic pigmentation would have independently occurred several times, at least within the genus *Drosophila* [42], [43]. If the same suppressive regulation of *bab* with sexually dimorphic pigmentation existed in *Diacamma* sp., *bab* expression patterns should be negatively correlated with those of *yellow*; however, there was no correlation between *bab* and *yellow* expressions (Text S1, Fig. S5). The female-specific *yellow* expression observed in *Diacamma* sp. was suggested to be regulated not by *bab,* but by another factor that affects the entire body.

In addition to body color, various traits exhibit sex differences in *Diacamma* sp., e.g., antennal shape, head morphology and wing polyphenism (Fig. 1A and B)[10]. The development of these traits must be regulated in temporally and spatially different ways. Determination of how developmental regulations are coordinated among traits will aid in the understanding of the proximate mechanisms of sexual dimorphism in social hymenopterans as well as in other animals.

## Materials and Methods

### Ants

*Diacamma* sp. is distributed throughout the Okinawa Islands in Japan, with the majority of the species' nests located underground. Colonies used in the present study were collected in Nakijin, Kenmin-no Mori (Onna), Hantagawa Park (Naha), Sueyoshi Park (Naha), and Seifa Utaki (Nanjo). Since the morphologically specialized queen caste has been secondarily lost in this genus, all females exhibit a worker phenotype; however, a single mated-worker (called a “gamergate”), which is capable of laying female-destined fertilized eggs, serves as a functional queen in each colony [44]. Gamergate-right colonies, each of which usually contains a gamergate with 50–200 workers, were reared in plastic containers (7.7×10.8×3.2 cm) filled with moistened plaster, maintained at 25°C under a 12L/12D photoperiod, and fed chopped mealworms (*Tenebrio molitor* larvae) three times a week.

No specific permits were required for the described field studies and the locations are not privately-owned or protected in any way. *Diacamma* sp. is not endangered or protected species.

### Evaluation of ant body color

Nineteen female and 26 male adults were collected from 9 colonies reared in laboratory. Specimens were sacrificed by placing them in tubes filled with ammonia gas and were subsequently placed on their sides on black drawing paper (4NCR-418, Lintec, Tokyo, Japan). Color images of each specimen were taken under constant light conditions using a CCD camera (DP50, Olympus, Tokyo, Japan) and were analyzed using Adobe Photoshop CS5 software (Adobe systems, San Jose, CA, USA). Three pixels were randomly chosen from images of each tergite and sternite of the third and fourth abdominal segments and color information for each pixel was determined using a HSB (hue, saturation, and brightness) color model, which defines colors in terms of three parameters: hue angle [45], saturation and brightness. Mean values of these parameters determined from results obtained from twelve pixels were used to define the indices of body color for each specimen.

### Observation of pigmentation processes during pupal periods

Prepupae were obtained from laboratory colonies and subsequently isolated and maintained in Easy Grip Culture Dishes (Falcon, 35×10 mm) at 25°C in constant darkness. Time course after pupation (shedding of larval cuticles) was recorded, and developmental stages of pupae were defined based on age in number of days post-pupation. Coloration of day 0, 3, 6, 9, 12, 15 and 18 pupae was recorded using a DP50 CCD camera (Olympus).

### Gene cloning

Total RNA of both male and female pupae was extracted using a SV total RNA isolation system (Promega, Madison, WI, USA). Extracted total RNA was reverse transcribed using Super Script III Reverse Transcriptase (Invitrogen, Carlsbad, CA, USA) with 50 pmol of oligo(dT)_20_ primer. Using the transcribed cDNA as a template, gene fragments of five orthologs (*pale*, *ddc*, *yellow*, *ebony*, and *tan*) were obtained through PCR amplification with gene-specific degenerate primers (Table S1) and *Takara Ex Taq* Hot Start Version (Takara, Shiga, Japan). PCR products were subcloned into pGEM-T vector Systems (Promega) and nucleotide sequences of each fragment were determined using ABI Prism Big Dye Terminator v3.1 Cycle Sequencing Kit in conjunction with a Model 3100 Genetic Analyzer (Applied Biosystems, Foster City, CA, USA). Nucleotide sequences were then subjected to a database search for homologs using the National Center for Biotechnology Information's (NCBI) BLAST X. Identified gene sequences were deposited in the GenBank/EMBL/DDBJ database.

### Quantitative PCR

Head-, thorax- and abdomen-derived total RNA was isolated from five pupae of each sex at each developmental stage (day 0, 3, 6, 9, 13, 15 and 18). Antennae and legs (excluding coxae) were removed from heads and thoraces, respectively. The thorax-abdomen boundary was defined as the boundary between the first and second abdominal segments, known as the propodeum and petiole, respectively. Although some pigmentation genes possess pleiotropic functions [22], [46], the contributions of these genes to pigmentation can be inferred by estimating their expression levels in the tissues where pigments are accumulated (i.e., the epidermis and cuticle). Therefore, total RNA was also extracted from the abdominal cuticle (including the epithelium) and the remaining parts of the abdomen (including the gut, nerves, and reproductive organs) of day 9 pupae of each sex.

Extracted RNA was reverse transcribed with High-Capacity cDNA Reverse Transcription Kits (Applied Biosystems). Novel primers for target genes were designed with Primer Express (ver. 2.0.0, Applied Biosystems) (Table S1). The long type isoform of the *pale* ortholog was targeted by a specific primer set. Relative quantification of cDNA was performed using *Power* SYBR Green PCR Master Mix and an ABI PRISM 7000 Sequence Detection System (Applied Biosystems) in accordance with manufacturer’s instructions. Quantitative PCR was technically triplicated. Quantified gene expression levels were normalized to those of *28S ribosomal RNA* (*28S rRNA*) orthologs (Text S2).

### RNA interference for examination of gene function

*yellow* dsRNA was synthesized using a MEGAscript RNAi Kit (Ambion, Woodward Austin, TX, USA) and a novel primer set designed within the isolated *yellow* sequence (Table S2) in accordance with manufacturer’s instructions. *GFP* dsRNA was also synthesized using GFP pQBI-polII (Wako, Osaka, Japan) as a template for PCR in order to serve as a control. dsRNAs were eluted with PBS and 0.01% phenol red in order to confirm dsRNA diffusion. The prepared dsRNA solution was injected into pupae between the third and fourth abdominal segments with a microinjector (IM-4B, NARISHIGE, Tokyo, Japan) under a binocular microscope. Injection micropipettes were produced from glass capillaries (GD-1, NARISHIGE) prepared using a micropipette puller (PB-7, NARISHIGE).

In RNAi experimentation, preliminary experiments were performed and the most effective conditions were adopted for use in all subsequent experiments. Fourteen female pupae and 12 male pupae were injected with 10 μg of *yellow* dsRNA in 1 μl PBS; these injections were performed twice for each pupa at day 0 and 6 prior to the *yellow* expression peak. As a control, 1 μl PBS and 10 μg *GFP* dsRNA in 1 μl PBS were injected at day 0 and 6 into 14 female pupae and 8 or 7 male pupae, respectively. Whether injected individuals eclosed or not was recorded and body coloration was evaluated as described in “**Evaluation of ant body color**”. Individuals that failed to shed their pupal cuticle in part or in whole by themselves were photographed subsequent to removal of their pupal cuticles. Reduction of *yellow* expression levels observed in RNAi experiments was confirmed using quantitative RT-PCR (Text S2).

## Supporting Information

Figure S1 **Adult cuticle formation and pigmentation during pupal development in females (A-F) and males (G-L).** No differences in the schedules of apolysis and adult cuticle formation were observed between the sexes. Pupal cuticle (arrow) was separated between day 0 and 3 (A-B and G-H), followed by formation of adult cuticle (arrowhead) between day 3 and 6 (B-C and H-I). Males possessed an adult cuticle about three times thicker than that of females (F and L). Pigmentation occurred between day 9 and 12 in both sexes (D-E and J-K) and pigments were distributed in a sex-specific manner. In females, the outer half of the cuticle possessed a darker pigment (F), while in males, pigments were more broadly distributed. “e”, “mf” and “br” indicate epidermis, molting fluid, and bristle, respectively.
(TIF)

Figure S2 **Ratios of pigmentation gene expression within the cuticle.** Specimens were dissected and abdominal tissues were separated and classified as either cuticle or other. For each pigmentation gene, relative expression level in each tissue classification was quantified, and the ratio of expression within the cuticle relative to those in the other abdominal tissues was calculated for both females (black bar) and males (orange bar). The *yellow* gene exhibited its greatest expression levels in female cuticles, which suggested that *yellow* was involved in black pigment synthesis (A). The *ebony* gene also showed relatively higher expression levels in the cuticles of females than it did in those of males (B). The *tan*, *pale*, and *ddc* genes showed relatively high expression levels within male cuticles (C-E).
(TIF)

Figure S3 **Effects of RNAi on body color as determined using the HSB model.** Hue angle, saturation, and brightness of pupae injected with PBS, *GFP*-, and *yellow*-dsRNA were evaluated. Black bars indicate color indices in females injected with PBS or *GFP*-dsRNA, orange bars indicate those in males injected with PBS or *GFP*-dsRNA, and shaded bars indicate those in individuals of both sexes that were injected with *yellow*-dsRNA. Data was analyzed separately based on sex using a Tukey’s test. ‘*’ and ‘**’ indicate significance at *p*<0.05 and 0.01, respectively.
(TIF)

Figure S4 **Effects of RNAi on** ***yellow*** **expression levels.** Relative expression levels of *yellow* in pupae injected with *GFP*- and *yellow*-dsRNA were evaluated. Black bars indicate expression levels in females injected with *GFP*-dsRNA, orange bars indicate those in males injected with *GFP*-dsRNA, and shaded bars indicate those in individuals of both sexes that were injected with *yellow*-dsRNA. Expression levels of *yellow* were normalized using expression levels of *28S rRNA*. Number of biological samples is shown at the bottom of each bar. Data was analyzed separately based on sex using a student’s *t*-test. ‘*’ indicates significance at *p*<0.05.
(TIF)

Figure S5 **Expression profiles of** ***bab*** **orthologs during pupal development of females (black lines) and males (orange lines).** *bab* expression levels in the head, thorax, and abdomen of each sex were quantified through quantitative RT-PCR. Expression levels were normalized to those of *28S rRNA*. Mean ± SD, n = 3 (technical triplication).
(TIF)

Table S1 **Primers used in gene cloning and quantitative PCR, lengths of cloned cDNA fragments and accession numbers.**
(DOC)

Table S2 **Primers used in dsRNA synthesis and quantitative RT-PCR of** ***yellow*** **and lengths of targeted cDNA fragments.**
(DOC)

Text S1 **Results of histological examinations of pigmentation process, evaluations of RNAi effects and expression analyses of** ***bab*** **.**
(DOC)

Text S2 **Methods of histological examinations, selection of an endogenous control gene for quantitative PCR, evaluations of RNAi effects, and expression analyses of** ***bric-á-brac*** **ortholog.**
(DOC)
