# Supplementary figures and images for: Sexually Dimorphic Body Color Is Regulated by Sex-Specific Expression of Yellow Gene in Ponerine Ant, Diacamma Sp
Source: PLoS One. 2014 Mar 25;9(3):e92875. doi: 10.1371/journal.pone.0092875 (PMC3965500; doi:10.1371/journal.pone.0092875)

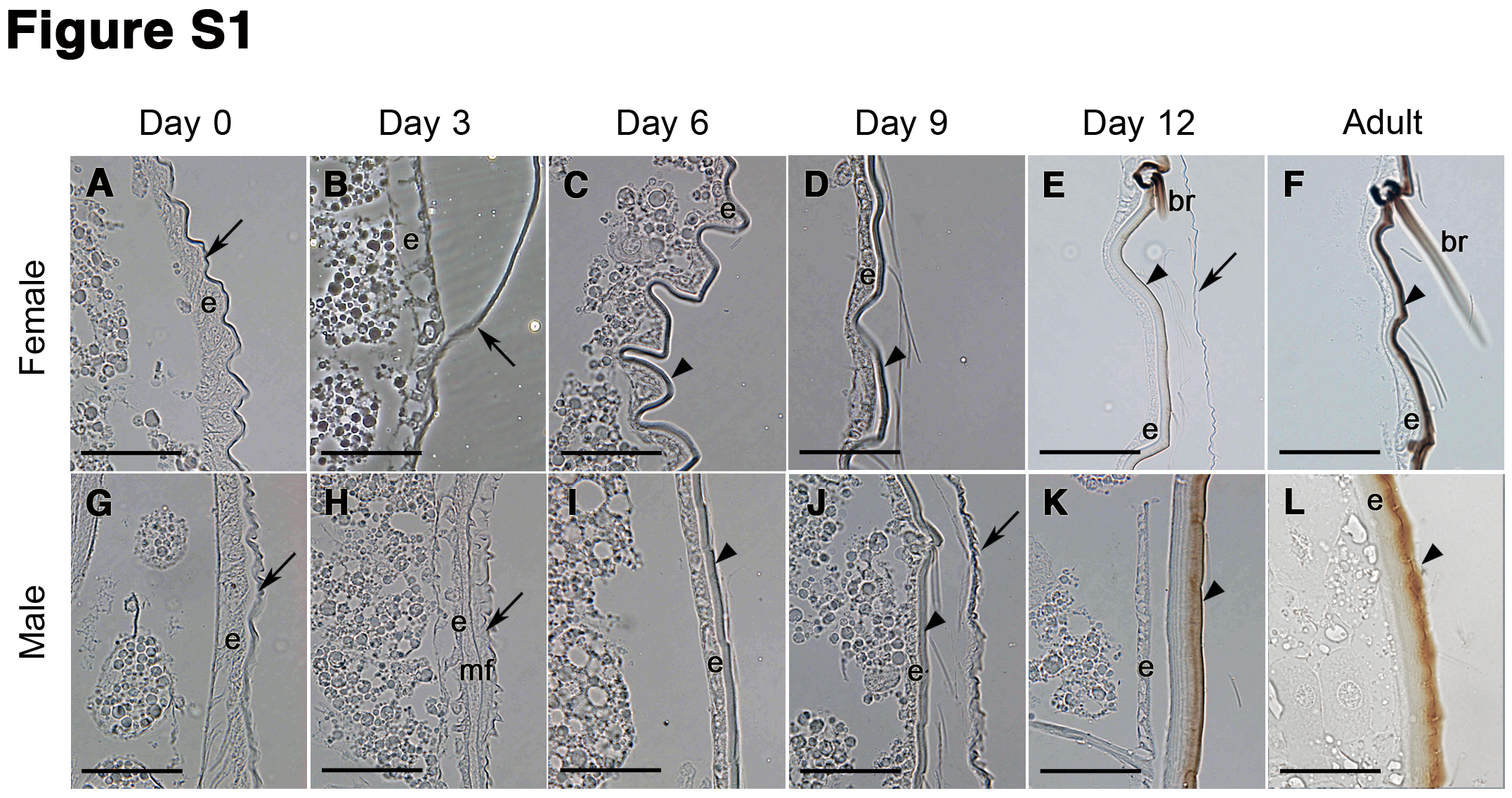

Supplement: Figure S1 — Adult cuticle formation and pigmentation during pupal development in females (A-F) and males (G-L). No differences in the schedules of apolysis and adult cuticle formation were observed between the sexes. Pupal cuticle (arrow) was separated between day 0 and 3 (A-B and G-H), followed by formation of adult cuticle (arrowhead) between day 3 and 6 (B-C and H-I). Males possessed an adult cuticle about three times thicker than that of females (F and L). Pigmentation occurred between day 9 and 12 in both sexes (D-E and J-K) and pigments were distributed in a sex-specific manner. In females, the outer half of the cuticle possessed a darker pigment (F), while in males, pigments were more broadly distributed. “e”, “mf” and “br” indicate epidermis, molting fluid, and bristle, respectively. (TIF) [file pone.0092875.s001.tif]

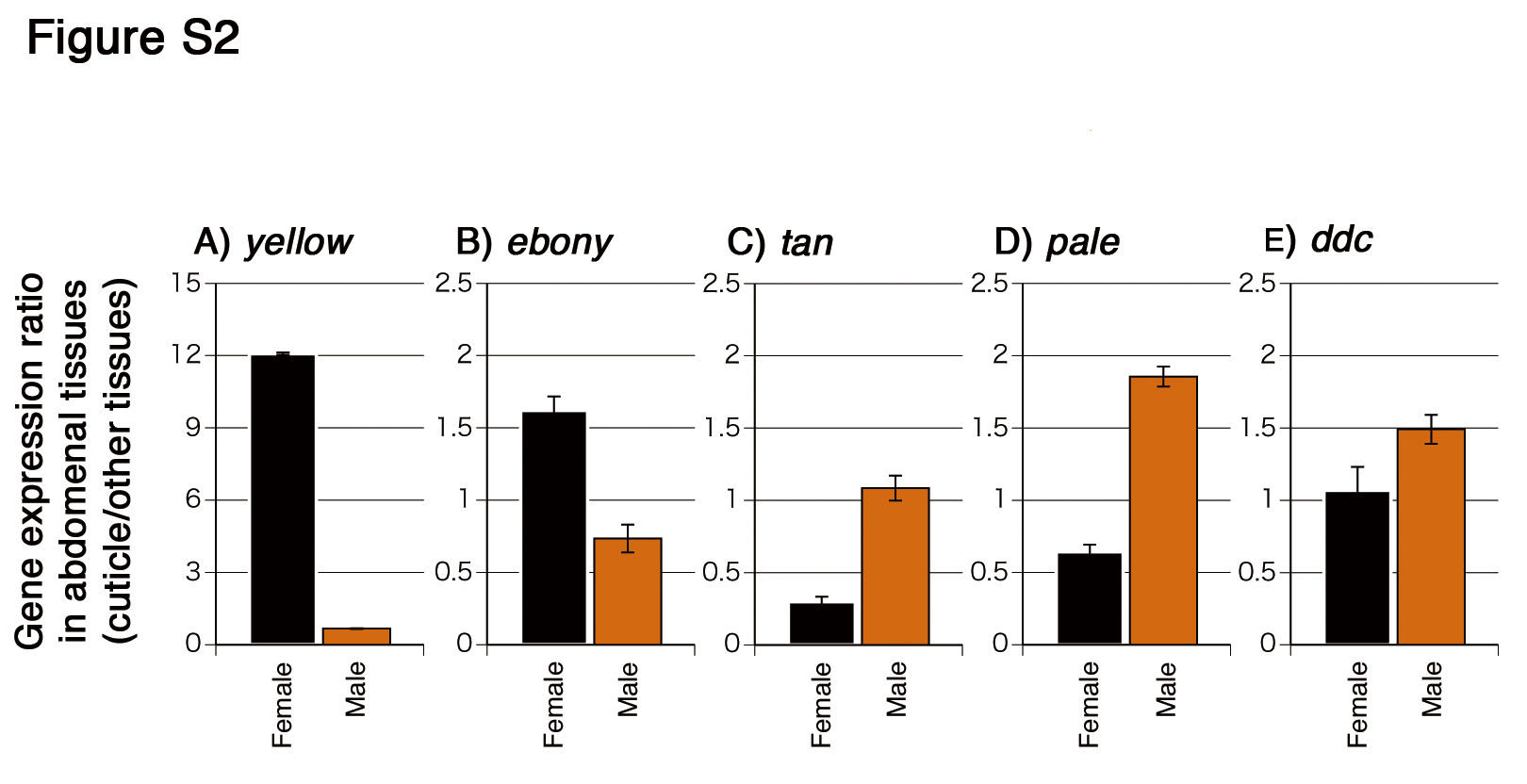

Supplement: Figure S2 — Ratios of pigmentation gene expression within the cuticle. Specimens were dissected and abdominal tissues were separated and classified as either cuticle or other. For each pigmentation gene, relative expression level in each tissue classification was quantified, and the ratio of expression within the cuticle relative to those in the other abdominal tissues was calculated for both females (black bar) and males (orange bar). The yellow gene exhibited its greatest expression levels in female cuticles, which suggested that yellow was involved in black pigment synthesis (A). The ebony gene also showed relatively higher expression levels in the cuticles of females than it did in those of males (B). The tan, pale, and ddc genes showed relatively high expression levels within male cuticles (C-E). (TIF) [file pone.0092875.s002.tif]

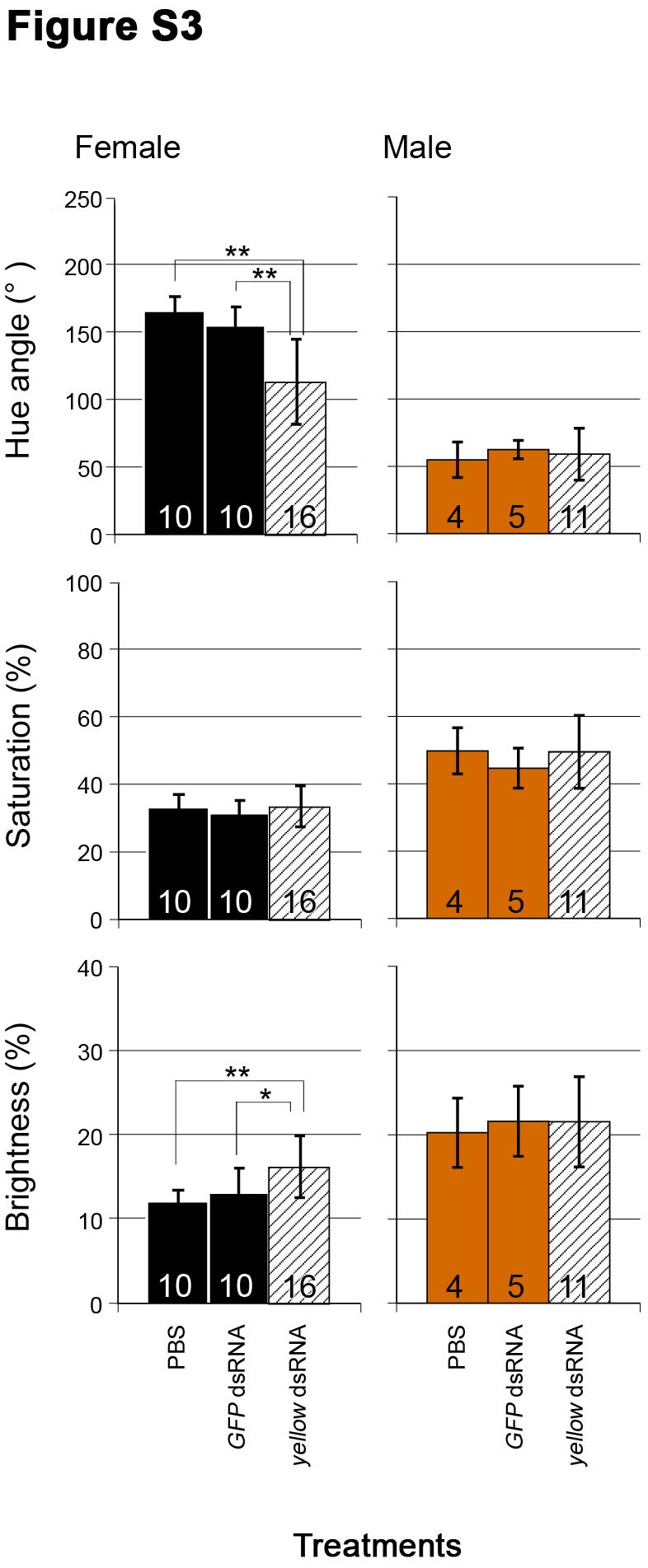

Supplement: Figure S3 — Effects of RNAi on body color as determined using the HSB model. Hue angle, saturation, and brightness of pupae injected with PBS, GFP-, and yellow-dsRNA were evaluated. Black bars indicate color indices in females injected with PBS or GFP-dsRNA, orange bars indicate those in males injected with PBS or GFP-dsRNA, and shaded bars indicate those in individuals of both sexes that were injected with yellow-dsRNA. Data was analyzed separately based on sex using a Tukey’s test. ‘*’ and ‘**’ indicate significance at p<0.05 and 0.01, respectively. (TIF) [file pone.0092875.s003.tif]

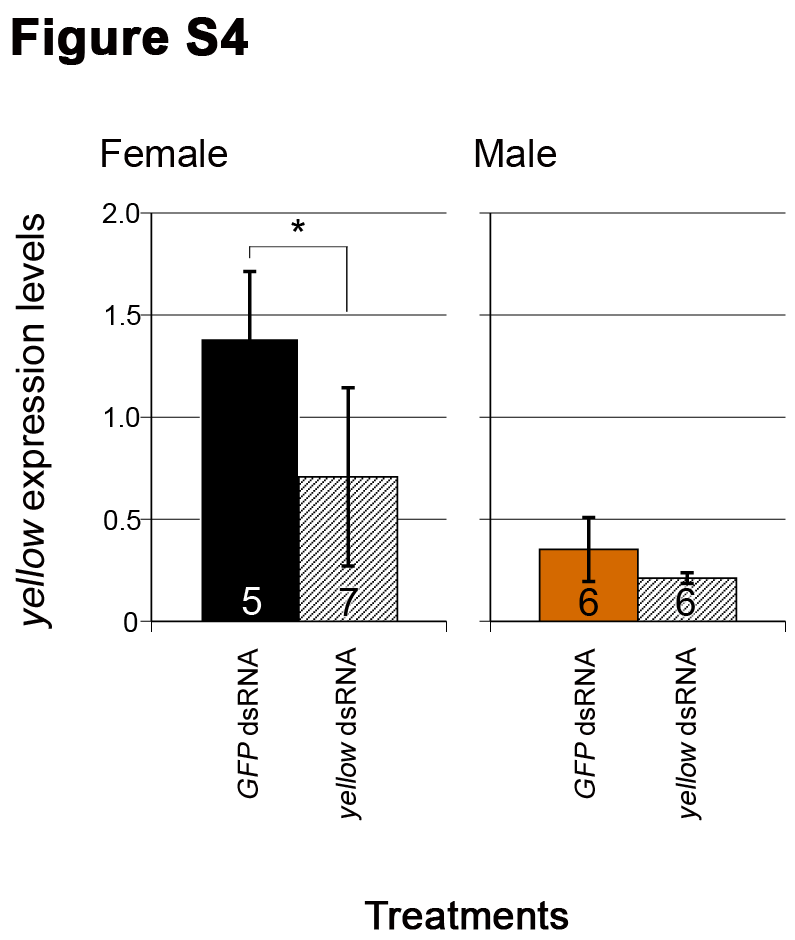

Supplement: Figure S4 — Effects of RNAi on yellow expression levels. Relative expression levels of yellow in pupae injected with GFP- and yellow-dsRNA were evaluated. Black bars indicate expression levels in females injected with GFP-dsRNA, orange bars indicate those in males injected with GFP-dsRNA, and shaded bars indicate those in individuals of both sexes that were injected with yellow-dsRNA. Expression levels of yellow were normalized using expression levels of 28S rRNA. Number of biological samples is shown at the bottom of each bar. Data was analyzed separately based on sex using a student’s t-test. ‘*’ indicates significance at p<0.05. (TIF) [file pone.0092875.s004.tif]

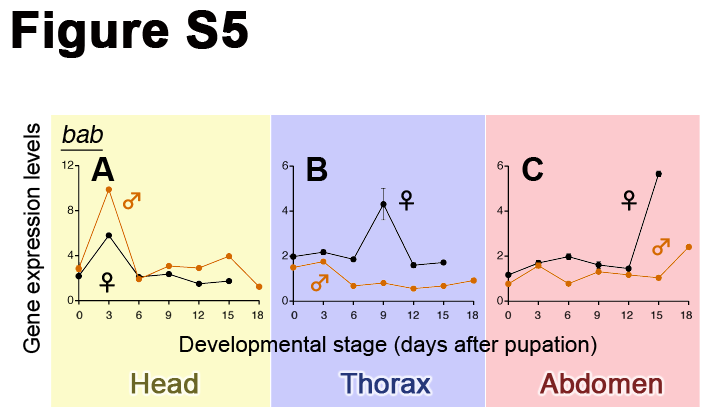

Supplement: Figure S5 — Expression profiles of bab orthologs during pupal development of females (black lines) and males (orange lines). bab expression levels in the head, thorax, and abdomen of each sex were quantified through quantitative RT-PCR. Expression levels were normalized to those of 28S rRNA. Mean ± SD, n = 3 (technical triplication). (TIF) [file pone.0092875.s005.tif]
