# Supplementary material for: Sexually Dimorphic Body Color Is Regulated by Sex-Specific Expression of Yellow Gene in Ponerine Ant, Diacamma Sp
Source: PLoS One. 2014 Mar 25;9(3):e92875. doi: 10.1371/journal.pone.0092875 (PMC3965500; doi:10.1371/journal.pone.0092875)
Supplement: Table S1 — Primers used in gene cloning and quantitative PCR, lengths of cloned cDNA fragments and accession numbers. (DOC) [file pone.0092875.s006.doc]

**Table S1.**

Primers used in gene cloning and quantitative PCR, lengths of cloned cDNA fragments and accession numbers.

| Target genes (Accession number) | Fragment length (bp) | Degenerate primers for gene cloning | Primers for quantitative RT-PCR |
| --- | --- | --- | --- |
| *pale (*AB907618; AB907617*)* | 532; 718 | F: 5’-CGYGARATGTTCGCNATCAAGAA-3’ | F: 5’-GACGATGCCCGTTTCGAA-3’ |
|  |  | R: 5’-GCGAAWCCRGGRTGRTTCATGTC-3’ | R: 5’-GCCCGTTGGCGAGCTT-3’ |
| *ddc* (AB907619) | 458 | F: 5’-GGNGGMGGTGTNATNSAGGG-3’ | F: 5’-CTGGGCACCACCAATTCC-3’ |
|  |  | R: 5’-AYYAGCATCCATTTRTGNGSATTGGA-3’ | R: 5’-TCGCCACGACGCCTATCT-3’ |
| *yellow* (AB907614) | 784 | F: 5’-ATACCGGAGAACGCDYTGCCCGTTGG-3’ | F: 5’-CTCAGACCGCAAGACATCAATC-3’ |
|  |  | R: 5’-CCAGCAGCCGACHGCATTYTGGTCGAT-3’ | R: 5’-CGCCGCTGCCCAGAT-3’ |
| *ebony* (AB907615) | 652 | F: 5’-TTYTAYGARCTKGGMGGSAAYTC-3’ | F: 5’-CTCTCGTGGAGAAGGGTCTGA-3’ |
|  |  | R: 5’-CMAGYTGCTGNGTYARNGG-3’ | R: 5’-AGCGACACCGATCGTTTTG-3’ |
| *tan* (AB907616) | 671 | F: 5’-ACACNAGNGGAACTCAYTAYGANRTTGG-3’ | F: 5’-TGTTGAGGCCGACCATAGAGA-3’ |
|  |  | R: 5’-GGNCCMAYTTCMRCRTTATGGAACA-3’ | R: 5’-GTGTAGCCCGGCAGGAAA-3’ |
| *β-actin* (AB510469) | 229 | F: 5'-CYATYGGYAAYGARAGRTTCCGTTG-3' | F: 5’-GCTAAGGCAGTGATCTCCTTCTG-3’ |
|  |  | R: 5'-TATTTCYTYTCRGGTGGHRCGATGAT-3' | R: 5’-GGCGGCACCACGATGTA-3’ |
| *gapdh* (AB907621) | 472 | F: 5’-GTNGCYRTYAAYGAYCCNTTCAT-3’ | F: 5’-TTACGATCCGAGCTGCAAAGT-3’ |
|  |  | R: 5’-CACGNCCRTCNCGCCA-3’ | R: 5’-CGAGAGGCCCCAAGCA-3’ |
| *28S rRNA* (AB908272) | 339 | - | F: 5’-TGTTGACGCAATGTGATTTCTG-3’ |
|  |  |  | R: 5’-CCGCGCTTGCTTGAATTT-3’ |
