# Supplementary material for: Sexually Dimorphic Body Color Is Regulated by Sex-Specific Expression of Yellow Gene in Ponerine Ant, Diacamma Sp
Source: PLoS One. 2014 Mar 25;9(3):e92875. doi: 10.1371/journal.pone.0092875 (PMC3965500; doi:10.1371/journal.pone.0092875)
Supplement: Table S2 — Primers used in dsRNA synthesis and quantitative RT-PCR of yellow and lengths of targeted cDNA fragments. (DOC) [file pone.0092875.s007.doc]

**Table S2.**

Primers used in dsRNA synthesis and quantitative RT-PCR of *yellow* and lengths of targeted cDNA fragments.

| Target genes | Fragment length (bp) | Primers for dsRNA synthesis | Primers for quantitative RT-PCR |
| --- | --- | --- | --- |
| *yellow* | 666 | F: 5’-GCGGATCACCCACGCTTACA-3’ | F: 5’-GAGATCTGGAGGAACAAGCTCTTC-3’ |
|  |  | R: 5’-CCAGCAGCCGACHGCATTYTGGTCGAT-3’ | R: 5’-ATTCAATGTGGCCGGTATCC-3’ |
