# Supplementary material for: Sexually Dimorphic Body Color Is Regulated by Sex-Specific Expression of Yellow Gene in Ponerine Ant, Diacamma Sp
Source: PLoS One. 2014 Mar 25;9(3):e92875. doi: 10.1371/journal.pone.0092875 (PMC3965500; doi:10.1371/journal.pone.0092875)
Supplement: Text S1 — Results of histological examinations of pigmentation process, evaluations of RNAi effects and expression analyses of bab . (DOC) [file pone.0092875.s008.doc]

**Text S1.** Results of histological examinations of pigmentation processes, evaluations of RNAi effects and expression analyses of the *bric-á-brac* ortholog.

**Results**

**Histological observation of the pigmentation processes**

Histological examination of pupal abdomens revealed the processes of both adult cuticle formation and pigment accumulation, and also revealed sex differences in cuticle properties (Fig. S1). In both sexes, the pupal cuticle had separated from the epidermal tissues by day 3 (apolysis, Fig. S1B and H), adult cuticle formation had commenced under the pupal cuticle between day 3 and 6 (Fig. S1C and I), and pigment had accumulated along the outer side of the adult cuticle (most likely the exocuticle) between day 9 and 12 (Fig. S1E and K). Immediately after eclosion, female cuticle was thinner (approximately 5 µm in thickness) and its surface was grooved, whereas male cuticle was thicker (approximately 15 µm in thickness) and its surface was smooth (Fig. S1F and L). Dark color pigments had accumulated in half of the outer cuticle in females, while lighter color pigments had accumulated in approximately one-third of the outer cuticle in males (Fig. S1F and L).

**Evaluation of the reduced expression levels of *yellow***

Comparison of *yellow* expression levels in pupae injected with *GFP-* and *yellow*-dsRNA determined that *yellow* RNAi decreased *yellow* expression by 49.0% in females and by 40.0% in males (Fig. S4). Reductions observed in females were significant (*p* = 0.02, Student’s *t*-test), while those observed in males were not (*p* = 0.08, Student’s *t*-test). Probably because *yellow* expression in males was very low, less reduction in the expression was expected upon *yellow* RNAi in males, as shown in Fig. S4.

**Expression levels of *bric-á-brac* during pupal development**

The partial sequences of *bab* ortholog (154 bp, accession number: AB907620) was obtained and its spatiotemporal expression pattern was examined. Unlike the *yellow* expression patterns (Fig. 3A-C), the *bab* expression patterns in both sexes varied among body parts (Fig. S5). Throughout the pupal developmental stages, the expression levels in heads were higher in males (Fig. S5A) while those in both thoraces and abdomens were higher in females (Fig. S5B and C). And then, the correlation between *bab* and *yellow* expressions was not statistically significant (*r* = 0.19, *p* = 0.24, a Spearman’s correlation coefficient by rank test). These results did not support the regulatory relationship of *bab* to *yellow* expression in *Diacamma* sp.
