# Supplementary material for: Sexually Dimorphic Body Color Is Regulated by Sex-Specific Expression of Yellow Gene in Ponerine Ant, Diacamma Sp
Source: PLoS One. 2014 Mar 25;9(3):e92875. doi: 10.1371/journal.pone.0092875 (PMC3965500; doi:10.1371/journal.pone.0092875)
Supplement: Text S2 — Methods of histological examinations, selection of an endogenous control gene for quantitative PCR, evaluations of RNAi effects, and expression analyses of bric-á-brac ortholog. (DOC) [file pone.0092875.s009.doc]

**Text S2.** Methods of histological examinations, selection of an endogenous control gene for quantitative PCR, evaluations of RNAi effects, and expression analyses of the *bric-á-brac* ortholog.

**Materials and Methods**

**Histological examination of pigmentation processes**

Several pupae at each stage of pupal development were fixed in FAA fixative (formalin : acetic acid : ethanol = 6 : 1 : 16) for 1 day prior to paraffin-embedding and processing into semi-thin sections (5 µm in thickness) using the methodology described previously ; however, the staining step was omitted, in order to observe pigment accumulation within the cuticle. Observations were conducted using a BX-51 microscope (Olympus) equipped with a DP50 CCD camera (Olympus) and the Viewfinder Lite program (Olympus).

**Selection of an endogenous control gene for quantitative PCR**

For candidates of endogenous control genes that were constitutively expressed, *Diacamma β-actin* , *glyceraldehyde-3-phosphate dehydrogenase* (*gapdh*) and *28S ribosomal RNA* (*28S rRNA*) orthologs were used (accession numbers are listed in Table S1). The *gapdh* was subcloned from *Diacamma* sp. as described in “**Gene Cloning**”. As for *28S rRNA*, gene sequence was obtained in previously performed gene-screening, Differential Display . In this method, total RNA was reverse transcribed with Bam-TG primer (CCCGGATCCT_(15)_ G), and PCR amplification was performed with Bam-TG primer and Hind III #14 primer (CGGGAAGCTTATTGGATTGGTC).

In order to select an endogenous control gene, expression levels of the three candidates were quantified using the primers detailed in Table S1, and expression stabilities were subsequently evaluated using geNorm and NormFinder . Results of these analyses indicated that *28S rRNA* was the most appropriate endogenous control.

**Evaluation of the reduced expression levels of *yellow***

For each of the RNAi and control experiments, 5-7 pupae were injected with either *yellow*-dsRNA or *GFP*-dsRNA. Total RNA was then extracted from the abdomen of each pupae at day 9 using the SV total RNA isolation system and subsequently reverse transcribed using High-Capacity cDNA Reverse Transcription Kits with 50 pmol of oligo(dT)_12-18_ primer. For qPCR, another primer set was designed within the isolated *yellow* fragment upstream to the region from which dsRNA was synthesized (Table S2). qPCR, which was carried out using each cDNA was biologically replicated (n = 5-7) and technically duplicated.

**Cloning and quantitative PCR of *bric-à-brac* ortholog**

Since the transcription factor Bric-à-brac negatively regulates the *yellow* expression in *Drosophila melanogaster* , the regulatory relationship of *bric-à-brac* (*bab*) ortholog with *yellow* in *Diacamma* sp. was examined. First, *bab* was subcloned from *Diacamma* sp. using the degenerate primers (forward primer 5’-CARTWYTGYCTRCGSTGGAACAA-3’ and reverse primer 5’-AYRATNGGRTGCTKGCANGG-3’) as described in “**Gene Cloning**”. Subsequently, the spatiotemporal expression pattern of *bab* was determined using the cDNAs from heads, thoraces and abdomens at each sex at each developmental stage and the newly designed primers (forward primer 5’-CAGACCAATCTCACCAACGTCTT-3’ and reverse primer 5’-GACCATCTTGTGGGCCTTGA-3’) as described in “**quantitative PCR**”. Quantified *bab* expression levels were normalized to those of *28S rRNA* orthologs. The correlation of the expression levels between *yellow* and *bab* was statistically tested using a Spearman’s correlation coefficient by rank test.

**Reference**

1. Miyazaki S, Murakami T, Kubo T, Azuma N, Higashi S, et al. (2010) Ergatoid queen development in the ant *Myrmecina nipponica*: modular and heterochronic regulation of caste differentiation. Proc Biol Sci 277: 1953-1961.

2. Okada Y, Miyazaki S, Miyakawa H, Ishikawa A, Tsuji K, et al. (2010) Ovarian development and insulin-signaling pathways during reproductive differentiation in the queenless ponerine ant *Diacamma* sp. J Insect Physiol 56: 288-295.

3. Okada Y, Miyazaki S, Koshikawa S, Cornette R, Maekawa K, et al. (2010) Identification of a reproductive-specific, putative lipid transport protein gene in a queenless ponerine ant *Diacamma* sp. Naturwissenschaften 97: 971-979.

4. Vandesompele J, De Preter K, Pattyn F, Poppe B, Van Roy N, et al. (2002) Accurate normalization of real-time quantitative RT-PCR data by geometric averaging of multiple internal control genes. Genome Biol 3: RESEARCH0034.

5. Pattyn F, Speleman F, De Paepe A, Vandesompele J (2003) RTPrimerDB: the Real-Time PCR primer and probe database. Nucleic Acids Res 31: 122-123.

6. Andersen CL, Jensen JL, Ørntoft TF (2004) Normalization of real-time quantitative reverse transcription-PCR data: a model-based variance estimation approach to identify genes suited for normalization, applied to bladder and colon cancer data sets. Cancer Research 64: 5245-5250.

7. Jeong S, Rokas A, Carroll SB (2006) Regulation of body pigmentation by the Abdominal-B Hox protein and its gain and loss in *Drosophila* evolution. Cell 125: 1387-1399.
